# Management of Bladder Cancer following Solid Organ Transplantation

**DOI:** 10.1155/2011/256985

**Published:** 2011-04-18

**Authors:** Jeffrey J. Tomaszewski, Jeffrey A. Larson, Marc C. Smaldone, Matthew H. Hayn, Stephen V. Jackman

**Affiliations:** Department of Urology, School of Medicine, University of Pittsburgh, Pittsburgh, PA 15213, USA

## Abstract

*Objective*. Present our experience managing bladder cancer following liver and renal transplantation. *Methods*. Single institution retrospective review of patients diagnosed with bladder urothelial carcinoma (BUC) following solid organ transplantation between January 1992 and December 2007. *Results*. Of the 2,925 renal and 2,761 liver transplant recipients reviewed, we identified eleven patients (0.2%) following transplant diagnosed with BUC. Two patients with low grade T1 TCC were managed by TURBT. Three patients with CIS and one patient with T1 low grade BUC were treated by TURBT and adjuvant BCG. All four are alive and free of recurrence at a mean follow-up of 51 ± 22 months. One patient with T1 high grade BUC underwent radical cystectomy and remains disease free with a follow-up of 98 months. Muscle invasive TCC was diagnosed in four patients at a median of 3.6 years following transplantation. Two patients are recurrence free at 24 and 36 months following radical cystectomy. Urinary diversion and palliative XRT were performed in one patient with un-resectable disease. *Conclusions*. Bladder cancer is uncommon following renal and liver transplantation, but it can be managed successfully with local and/or extirpative therapy. The use of intravesical BCG is possible in select immunosuppressed patients.

## 1. Introduction

Renal and liver transplantation are the optimal treatments for end-stage renal and liver disease [1, 2]. While the development of bladder cancer following renal and liver transplantation is quite rare [3], transplant recipients have an increased incidence of bladder cancer [4, 5], ranging from 0.08% to 2.1% [6, 7], and frequently present with higher-stage bladder cancer compared to the general population [5]. Optimal management of bladder cancer among renal and liver transplant recipients is not well defined. Most management strategies have only been briefly described in anecdotal case reports [8] and retrospective series [7]. We present our single institution experience managing bladder cancer after renal and liver transplantation.

## 2. Patients and Methods

Through an Institutional Review Board approved protocol, we retrospectively reviewed our institutional transplant database to identify patients diagnosed with bladder urothelial carcinoma (BUC) following renal and liver transplantation between January 1992 and December 2007. We examined demographic information including gender, age at transplant and cancer diagnosis, type of immunosuppression, smoking history, and time to cancer diagnosis. Tumor characteristics, including clinical and pathologic stage, mode of presentation, treatment (including adjuvant therapy and urinary tract reconstruction), and tumor progression were also evaluated. Postoperative outcomes reviewed include disease recurrence, allograft function following treatment, overall and cancer-specific survival, and length of followup. 

No patient had a history of bladder cancer or hematuria prior to transplantation. Transurethral resection of bladder tumor (TURBT) with or without intravesical immunotherapy or chemotherapy (one patient received mitomycin-C) was used for treating nonmuscle-invasive tumors. Patients were treated in accordance with the AUA guideline for the management of nonmuscle invasive bladder cancer (Hall MC, J Urol, 2007). BCG was used in select patients, and criteria for consideration of BCG included patients at high risk for recurrence, large tumors, multifocal tumors, high-grade disease, or presence of CIS. Surgeon preference also influenced the use of intravesical BCG, as multiple different urologists provided treatment to patients in the current series. BCG was administered as described by Lamm et al. Radical cystectomy with urinary diversion or palliative external beam radiotherapy was used for those with muscle invasive disease. None of the patients with muscle invasive disease received neoadjuvant or adjuvant therapies. The radiation field was not significantly altered in renal transplant patients, and immunosuppressive regimens remained unchanged in all patients. Patients were followed postoperatively at regular intervals per established surveillance protocols.

## 3. Results

Of the 2,925 renal and 2,761 liver transplant recipients reviewed, we identified eight patients following kidney transplant and three patients following liver transplant (*n* = 11, 0.2%) who developed bladder cancer. The mean age at transplantation was 62 ± 13 years (range 38–82 years) and all were male. Bladder cancer diagnosis was made at a mean interval of 39 ± 24 months (range 3.5–76 months) following transplantation. Mean age at the time of diagnosis was 65 ± 12 years (range 44–83), and mean followup was 40 ± 27 months (range 12–98 months). Maintenance immunotherapy protocols included tacrolimus + prednisone, tacrolimus + mycophenolate mofetil + prednisone, tacrolimus + mycophenolate mofetil, and tacrolimus alone in 54.5%, 27.3%, 9.1%, and 9.1% of patients, respectively. It was not necessary to alter immunotherapy protocols following diagnosis or in the treatment of BUC. There were no deleterious effects on graft survival or function following urologic intervention. Prior to urologic intervention, mean serum creatinine was 1.4 mg/dL (range 0.9–2.0 mg/dL). At last followup, serum electrolyte levels were normal, and mean serum creatinine level was 1.5 ± 0.4 mg/dL (range 1.0–2.1 mg/dL). Liver function tests were not affected by urologic intervention. 

Ten patients presented with gross hematuria, and one patient was diagnosed incidentally by cross-sectional imaging. Imaging studies performed at the time of diagnosis revealed no evidence of lymphadenopathy, metastasis, or upper tract urothelial carcinoma. Two patients with low-grade T1 BUC were managed by transurethral resection (TURBT) alone. Three patients with CIS and one patient with T1 BUC were treated by TURBT and adjuvant intravesical bacille Calmette-Guerin (BCG) immunotherapy. The patient with T1 disease recurred locally after 7 months and was treated with a repeat course of BCG. Two of the patients with CIS recurred at 18 and 12 months: the first was managed with repeat TURBT and a 6-week course of Mitomycin C; the second received another 6-week course of BCG. All four patients treated with BCG are alive and free of further recurrence at a mean followup of 51 ± 22 (36–84) months. One patient with T1 high-grade BUC and a history of bilateral cutaneous ureterostomies (performed following transplantation for treatment of vesicoureteral fistula) underwent radical cystectomy and ureteroureterostomy and remains disease-free with a followup of 98 months. None of the patients with T1 disease progressed to muscle invasive disease (Table 1).

Muscle invasive transitional cell carcinoma was diagnosed in four patients at a median of 3.6 years (range 2 to 6 years) following transplantation (Table 2). Two patients underwent radical cystectomy with urinary diversion, one with orthotopic neobladder, and one with percutaneous allograft nephrostomy tube placement (due to significant scar tissue and adhesions limiting small bowel mobilization and creation of urinary diversion). Both patients remain recurrence-free at 24 and 36 months of followup, respectively. Ileal conduit urinary diversion and palliative radiation were performed in one patient with unresectable disease, who subsequently died from disseminated intravascular coagulation 16 months following cancer diagnosis. One patient received palliative XRT and died of diffuse liver and pulmonary metastases 12 months following treatment. Criteria for consideration of palliative XRT include evidence of metastases at the time of diagnosis of muscle invasive BUC and unresectable disease.

## 4. Discussion

The increased risk for the development of malignancies following renal and liver transplant is well documented [9]. Of special interest to urologists is the increased incidence of genitourinary malignancies, including kidney and bladder cancer, following transplantation [10]. Transplant recipients are up to 3.3-times more likely to develop BUC than the general population [4]. More alarmingly, recent data also suggest that renal transplant recipients and patients with end-stage renal disease present with higher-stage [5], biologically more aggressive tumors [11] and experience worse outcomes than the general population [11]. The etiology of increased risk for BUC among transplant recipients is multifactorial [5], and in addition to common risk factors for BUC such as smoking, also includes risk factors unique to the transplant recipient, such as direct cytotoxic damage from immunosuppressive agents [12], impaired DNA repair mechanisms in immunocompromised patients [13], impaired protection against viral oncogenes [14], and urinary tract infections [15]. 

The incidence of de novo BUC following renal and liver transplantation in our series is 0.27% and 0.11%, respectively, and is comparable to other series. The University of California, San Francisco, reported the development of de novo BUC in 0.08% of over 6000 renal transplant recipients [7] while The University of Wisconsin reported a 0.19% incidence of BUC after renal transplantation [4]. The United Network for Organ Sharing (UNOS) database (1986 to 2001) reported a similarly low prevalence (0.024%) of post-transplantation BUC [7]. Conversely, Kamal et al. [3] reported a higher rate (0.37%) of BUC following renal transplantation in Egypt than observed in the present cohort. The authors accounted for this discrepancy by the high prevalence of bilharzial infestation among the Egyptian population; and there was evidence of bilharzial cystitis in five of the seven cases reported by Kamal et al. [3]. 

As a result of perioperative and induction immunosuppressive therapy used in the immediate posttransplant period, transplant recipients experience the greatest risk of developing bladder cancer within the first 6 years after transplantation [11]. Numerous reports document the rapidly progressive nature of bladder cancer in the transplant population [7, 8]. Comparison of transplant recipients from the Israel Penn International Transplant Tumor Registry to adults in the general population (from the Surveillance, Epidemiology, and End Results database) revealed a more advanced cancer stage at diagnosis in transplant recipients as well as a worse stage-stratified disease-specific survival [11]. Immunosuppressive therapy alters the host-tumor relationship by inhibition of IL-2 stimulated T-cell proliferation, enhancement of tumor angiogenesis, and a dose-dependent reduction in DNA repair capability, resulting in increased biologic aggressiveness, and likely explains why transplantation confers a poor prognosis for cancer outcomes [11]. While most primary diagnoses of BUC in the general population are low-grade superficial disease [16], a majority of our cohort presented with high grade disease, and muscle invasion and nodal metastases were noted in 4 and 2 patients, respectively. Although recent data have demonstrated favorable outcomes utilizing neoadjuvant chemotherapy in patients with muscle-invasive bladder cancer, the patients in the current series were treated prior to the widespread utilization and acceptance of neoadjuvant chemotherapy [17]. Neoadjuvant cisplatin can be considered in solid organ transplant recipients [18], but no specific selection criteria exist as data remain limited; its use should be approached with the same caution one would use administering adjuvant chemotherapy to immunosuppressed transplant recipients. However, reasonable cancer-specific survival was achieved after aggressive therapy. The survival rates of 90% (pTxNxMx) and 50% (≥pT2) at a mean followup of 40 ± 27 months in our cohort are comparable to the 5-year overall survival observed in large series following cystectomy in nontransplant patients with pT2 and pT3a BUC (77% and 58%, resp.) [19]. Our findings suggest that early and aggressive screening should be considered in transplant recipients [5, 11]. 

To our knowledge, there are no published recommendations for the management of immunosuppression after surgical resection of BUC. However, some have suggested that for patients with localized disease, no alteration in immunosuppression is required [20]. The experience with adjuvant cisplatin-based chemotherapy in renal transplant recipients is limited, but it has been used in those with well functioning and poorly functioning allografts [7, 21]. Dose increase and the side effect profile are adversely affected because most immunosuppressive medications have systemic effects such as nephrotoxicity (cyclosporine) or bone marrow suppression (cyclophosphamide, mycophenolate) [7]. However, decrease of immunosuppressive medications is possible without rejection since the chemotherapeutic cytotoxic agents are themselves immunosuppressive and may prevent allograft rejection [7]. Some have argued that cyclosporine itself increases the risk of malignancy compared to other immunosuppressive medications and, therefore, should not be part of standard regimens [7]. Additional data are needed to support this concept. The risks of continued immunosuppression merely to preserve the allograft when faced with a known, aggressive malignancy are unclear and deserve investigation [7]. 

Hematuria was the main presenting symptom in the current study. While hematuria in renal transplant recipients can have several etiologies, it requires thorough evaluation with urinalysis and culture, upper urinary tract imaging, and cystoscopy [22]. Urinary cytology is reliable and has a significantly low false-positive rate in renal transplant recipients [23]. Newer diagnostic modalities such as fluorescent in-situ hybridization and tumor markers have not been evaluated in the transplant population [22]. While previously reported data have demonstrated an increased incidence (66.6%) of upper tract BUC in small series of renal transplant recipients with bladder tumors [24], no upper tract disease was identified in our cohort. Interestingly, none of the 11 patients who developed BUC had a previous smoking history, a well-recognized risk factor for the development of BUC. 

The optimum management and clinical outcomes of bladder cancer in renal and liver transplant recipients are not well defined, and treatment is dependent upon disease stage [22]. For patients with Ta or T1 low-grade disease, TURBT may be both diagnostic and therapeutic. The use of adjuvant intravesical therapies such as BCG and Mitomycin C should be considered for patients at high risk for recurrence (large tumors, multifocal tumors, high-grade disease, or presence of CIS) [22]. While BCG has been shown to be more effective than Mitomycin C to prevent recurrence and disease progression [36], its use in immunocompromised patients has been cautioned [4, 37]. BCG is a live attenuated strain of *Mycobacterium* bovis that acts as a recall antigen and evokes a nonspecific immune response [3], which carries the risk (1 in 12,500 patients) of severe tuberculous reaction, sepsis, and death among immunosuppressed patients [4, 37]. The efficacy of intravesical BCG in immunosuppressed patients has also been questioned. However systemic immunosuppression may not always induce a local immunosuppressive effect, and therefore the bladder immunologic-inflammatory reaction obtained with BCG may maintain its efficacy [27, 37]. While Buzzeo et al. [4] refrained from using BCG in immunosuppressed patients, Palou et al. [27] reported safe administration of intravesical BCG in 3 renal transplant recipients. It should be noted that while there were no adverse reactions to BCG, all 3 patients were treated with prophylactic isoniazid and rifampin [27]. The safe administration of intravesical BCG without concomitant antituberculin prophylaxis has also been reported in 3 immunosuppressed renal transplant recipients [3, 28]. In the present study, we demonstrate the safe and efficacious administration of adjuvant intravesical BCG therapy in 4 immunosuppressed transplant recipients. Caution is advised prior to administration of intravesical therapy, as mortality due to general visceral deficiency has been reported in a transplant recipient following BCG treatment [38]. Given the limited data regarding the safety of intravesical BCG in immunosuppressed patients, we advocate its use only in select patients while maintaining a high degree of clinical suspicion for sepsis and administration of prophylactic antibiotics on a case by case basis. Ciprofloxacin was used in a single dose before each BCG treatment in 80% of our patients. 

 For patients with muscle-invasive disease, radical cystectomy offers the best chance at cure and is technically feasible in kidney and liver transplant recipients [7, 22]. Reported outcomes of radical cystectomy in renal transplant recipients are summarized in Table 3. It deserves mention that the rate of muscle invasive BUC observed in our cohort (36%) was higher than that typically seen in the general population. Although immunosuppressed transplant patients may be at higher risk to develop more aggressive disease, the current series is too small to draw definitive conclusions. Prolonged survival and graft preservation have been demonstrated after radical cystectomy in transplant recipients [3, 7]. Reconstructive options range from urinary diversion with incontinent (ileal conduit, cutaneous ureterostomy, nephrostomy) or continent urinary diversions (ileal neobladder). Ileal conduit urinary diversion, which reduces exposure of urine to the absorptive bowel mucosa, should be used in patients with significant renal dysfunction [22], while orthotopic substitution is feasible in patients with a creatinine clearance of >40 mL/min [3, 7, 25]. Radical cystectomy should be performed in appropriate situations, and in some patients with advanced disease, cystectomy may not be feasible. One patient in the cohort underwent palliative radiation and ileal conduit urinary diversion following aborted cystectomy, for the purpose of providing local symptom control. Radical cystectomy with urinary diversion did not impact on graft function or survival in the current cohort. 

Given the high incidence (up to 41%) [26] of synchronous upper tract TCC in renal transplant recipients, some advocate that prophylactic bilateral native nephroureterectomies be performed at the time of cystectomy [7, 26]. Lang et al. [25], conversely, found no evidence of upper tract TCC in 75% of patients undergoing prophylactic nephroureterectomy. Prophylactic nephroureterectomy was not performed in the current cohort, and followup studies to date reveal no evidence of metachronous upper tract disease. The allograft is rarely the source of BUC and in the vast majority of cases can be preserved [24]. Given the morbidity of nephroureterectomy in this complex population, we agree that prophylactic nephroureterectomy should be reserved for patients with high-risk features, such as documented multifocal disease and CIS. 

The current study is limited by sample size and retrospective design. Reporting bias may have also occurred in the early years of the study, prior to effective data acquisition. Additionally, although all patients were screened for bladder cancer prior to renal transplant, we cannot exclude the presence of a pre-existing tumor. Given the short preclinical latency of BUC [39], however, pre-existing tumors would likely have been detected at the time of ureteral stent removal in the early post-operative period. Therefore, given the short preclinical latency period and the thorough pre-transplant evaluation, factors associated with transplantation more likely contributed to tumorigenesis as opposed to increased surveillance in renal and liver transplant recipients. Additionally, the mean duration from renal transplant to diagnosis of bladder cancer was 3.3 years, and such a long period between renal transplantation and BUC development suggests that these tumors were not present prior to transplantation.

## 5. Conclusions

We report encouraging oncologic outcomes in eleven patients undergoing definitive therapy without compromising allograft function. The use of intravesical BCG is possible in select immunosuppressed patients with CIS or nonmuscle-invasive disease. Aggressive extirpative surgery and urinary diversion is technically feasible and should be considered in transplant recipients with muscle invasive BUC and a good performance status.

##  Conflict of Interests 

The authors declare that they have no conflict of interests to report.

## Figures and Tables

**Table 1:** Characteristics of patients with nonmuscle-invasive TCC of the bladder.

Patient number	1, 2	3	4	5	6	7
Age at transplant	66	77	82	66	63	45
Type of transplant	Liver	Renal	Renal	Renal	Liver	Renal
Age at diagnosis	66	79	83	72	65	47
Immunosuppression	FK, MMF, Pred	FK, Pred	FK	FK, MMF	FK, Pred	FK, Pred
Clinical stage	T1NxMx	T1NxMx	TisNxMx	TisNxMx	TisNxMx	T1N0Mx
Histology	TCC	TCC	CIS	CIS	CIS	TCC
Grade	Low	Low	High	High	High	High
Treatment	TURBT	TURBT	TURBT	TURBT	TURBT	Cystectomy, ureteroureterostomy
Adjuvant therapy	None	BCG	BCG	BCG	BCG	Mitomycin C
Followup (months)	28, 24	40	36	84	42	98
Recurrence	None	Local	Local	None	Local	None

FK: tacrolimus; MMF: mycophenolate mofetil; Pred: prednisone; CIS: carcinoma in site; TURBT: transurethral resection bladder tumor; BCG: bacille-Calmette Guerin.

**Table 2:** Characteristics of patients with muscle-invasive TCC of the bladder.

Patient number	1	2	3	4
Age at transplant	38	58	60	66
Type of transplant	Renal	Liver	Renal	Renal
Age at diagnosis	44	60	63	70
Immunosuppression	FK, Pred	FK, MMF, Pred	FK, Pred	FK, Pred
Pathologic stage	T3aN0M0	T3N2M0	T2N0M0	T2N0M1
Histology	TCC, CIS	TCC	TCC	TCC
Grade	High	High	High	High
Treatment	Cystectomy, allograft nephrostomy	Cystectomy, neobladder	Palliative XRT, Ileal conduit	Palliative XRT
Followup (months)	24	36	16	12
Metastasis (months)	None	None	None	Liver, pulmonary (12)
Status	Alive	Alive	Deceased (DIC)	Deceased

FK: tacrolimus; MMF: mycophenolate mofetil; Pred: prednisone; CIS: carcinoma in site; TURBT: transurethral resection of bladder tumor; DIC: disseminated intravascular coagulation; XRT: external beam radiotherapy.

**Table 3:** Summarized data on all cases of radical cystectomy and urinary diversion performed for postrenal transplant bladder cancer [3].

Study	Number of radical cystectomies	Type of urinary diversion (*n*)	Followup (months)	Oncologic outcome (*n*)	Graft function (*n*)
Present	3	Neobladder (1) Allograft nephrostomy (1) Cutaneous ureterostomies (1)	24–98	NED (3)	Stable (3)
Kamal et al. [3]	5	Neobladder (5)	3–24	NED (3) Mets (2)	Stable (5)
Lang et al. [25]	4	Neobladder (4)	11–118	NED (3) Local recurrence (1)	Stable (3) Chronic allograft nephropathy (1)
Master et al. [7]	3	Neobladder (2) IC (1)	10–105	NED (1) Ureteric recurrence (1) Paravaginal recurrence (1)	Stable (3)
Kao et al. [26]	4	IC (4)	4–39	NED (3) Mets (1)	Unknown
Palou et al. [27]	1	IC (1)	10	NED (1)	Stable (1)
Wang et al. [28]	0				
Giessing et al. [29]	1	Neobladder (1)	20	NED (1)	Stable (1)
Perabo and Schultze-Seemann [30]	1	Neobladder (1)	8	NED (1)	Stable (1)
Colombo et al. [31]	1	Neobladder (1)	8	NED (1)	Stable (1)
Schmidt et al. [32]	1	Unknown	Unknown	Unknown	Unknown
Lam et al. [33]	1	IC (1)	Unknown	Unknown	Unknown
Lemmers and Barry [34]	2	IC (2)	6–24	NED (2)	Stable (2)
Tuttle et al. [35]	1	IC (1)	14	NED (1)	Stable (2)

NED, no evidence of disease; IC, ileal conduit; Mets, metastases.
